# Quality Characterization and Anti-Fatigue Properties of Yak (*Bos grunniens*) Leather Gel in a Mouse Endurance Swimming Model

**DOI:** 10.3390/foods15203623

**Published:** 2026-10-12

**Authors:** Tengteng Zhang, Songshan Zhang, Xinyu Yang, Peng Xie, Mingwu Zang, Xinjun Zhang, Weiru Song, Rende Song, Yue Cui, Xiaoguang Gao

**Affiliations:** 1 College of Food Science and Biology, Hebei University of Science and Technology, No. 26 Yuxiang Street, Yuhua District, Shijiazhuang 050018, China; ztteng8364@163.com (T.Z.); xinyue0821@163.com (X.Y.);; 2 Institute of Animal Sciences, Chinese Academy of Agricultural Sciences, Beijing 100193, China; 3 Qinghai Xiahua Halal Meat Products Co., Ltd., Haibei Tibetan Autonomous Prefecture 812200, China; 4 Yushu Prefecture Animal Disease Prevention and Control Center, Yushu 815000, China

**Keywords:** yak by-products, yak leather gel, flavor characteristics, anti-fatigue, gut microbiome

## Abstract

As a major by-product of yak (*Bos grunniens*) processing, yak skin remains largely underutilized. In this study, a novel functional food, Yak Leather Gel (YLG), was developed, and its quality, flavor characteristics, and anti-fatigue activity were systematically evaluated with Colla Corii Asini (CCA) as a reference. YLG exhibited significantly higher iron and proline contents than those in CCA, suggesting a distinct nutritional potential. For flavor analysis, volatile compounds were assessed based on odor activity values (OAVs). In YLG, nine compounds were identified as key aroma contributors. YLG maintained typical baked notes while displaying characteristic nutty and smoky aromas, primarily derived from pyrazine compounds, which distinguished it from CCA. Importantly, YLG supplementation showed stronger anti-fatigue effects than CCA in an animal model. In mice, YLG significantly prolonged exhaustive swimming time, increased the levels of liver glycogen, glucose, superoxide dismutase (SOD), glutathione peroxidase (GSH-Px), and ATP, and decreased blood malondialdehyde (MDA) and lactate dehydrogenase (LDH). These biochemical changes indicated enhanced energy metabolism and reduced oxidative stress. Furthermore, YLG increased the relative abundance of exercise-related beneficial gut microbiota. These findings indicate that YLG exhibits promising anti-fatigue activity compared with CCA, which may be linked to improved energy metabolism, enhanced antioxidant capacity, and beneficial modulation of gut microbiota. The findings support the value-added utilization of yak skin as a high-quality functional food ingredient, providing a theoretical basis for further product development.

## 1. Introduction

The Yak (*Bos grunniens*) is a unique species native to the Qinghai-Tibet Plateau and surrounding mountainous areas, known for its high altitude habitat, unpolluted environment, and seasonal variations [1]. Currently, the processing technologies for yak meat and milk are well established, while yak skin, a byproduct of yak-related products, is primarily utilized in the leather industry and remains underutilized in other areas [2]. The conversion of yak hide into high-value functional ingredients is therefore an attractive strategy to improve the economic benefits of the yak industry and reduce resource waste. Animal skin accounts for a specific proportion of animal live weight (4–12%) and is considered one of the most valuable by-products, with potential for processing into various products. The fresh yak hide, after removing moisture, contains crude protein (89.74%), fat (5.27%), and ash and other components (5.99%) by dry weight. Collagen makes up 85% of the crude protein, being the main nutritional component of yak hide [3].

Yak Leather Gel (YLG), also known as yak skin glue, with the Chinese name máo xī jiāo, is prepared from fresh yak skin. In this process, acid and alkali degrade collagen fiber molecules by disrupting intermolecular ionic crosslinks and hydrogen bonds, allowing the complete release of collagen molecules. The mixture is subsequently heated to break the hydrogen bonds while preserving the core triple-helical conformation of collagen, resulting in a dual adhesive agent after purification [3]. YLG is composed of 18 types of amino acids, and two kinds of trace elements [1].

Ejiao, also known as donkey-hide gelatin (Colla Corii Asini, CCA), is a traditional Chinese medicinal material prepared primarily by decocting and concentrating the gelatin extracted from soaked donkey skin [4,5]. The primary chemical constituents of CCA are amino acids, trace elements, polysaccharides, and other bioactive compounds [6,7]. Although CCA has long been recognized for its tonic effects, its supply is limited by the scarcity of donkey resources and high production costs. Whether YLG can achieve comparable or even better effects than CCA on exercise performance and gut microbial composition, however, remains poorly understood.

Although previous studies have demonstrated that yak skin-derived collagen peptides possess antioxidant and potential health-promoting activities, most investigations have focused on the extraction, structural characterization, or in vitro functional properties of yak skin-derived components. The physiological effects of YLG, a traditional gelatin product directly derived from yak skin, have received much less attention, and whether it can improve exercise endurance and alleviate fatigue in vivo remains to be determined.

Fatigue is a multifaceted physiological and psychological condition resulting from factors such as physical exertion, mental overload, or insufficient rest. Fatigue impairs both cognitive and physical performance, and if not effectively managed, can progress to chronic fatigue syndrome, which exerts profound detrimental effects on overall physiological functioning. The pathogenesis of fatigue remains incompletely elucidated, but three major mechanistic hypotheses are widely accepted: energy depletion (consumption of ATP, glycogen, and lipid reserves), accumulation of fatigue-inducing metabolites (lactic acid, pyruvate, and serum urea nitrogen), and oxidative stress mediated by reactive oxygen species (ROS), including hydroxyl radicals, superoxide anions, and hydrogen peroxide [8,9]. At present, there has been growing scientific interest the anti-fatigue effect and mechanism of natural bioactive compounds. Peptides derived from yak by-products significantly prolonged the swimming duration of mice, indicating their potential anti-fatigue effects [8]. Nevertheless, a systematic comparison of the nutritional, flavor and anti-fatigue properties of YLG and CCA is still lacking. We therefore hypothesized that YLG supplementation could exert anti-fatigue effects by improving energy metabolism, enhancing antioxidant defense, maintaining skeletal muscle integrity, and altering gut microbiota composition.

Therefore, this study aimed to systematically evaluate the nutritional characteristics, flavor profiles, and anti-fatigue effects of YLG compared with CCA. To this end the physical properties and flavor-active compounds of gelatin derived from yak hide and donkey hide were first compared. The anti-fatigue physiological functions were then explored through mouse experiments, with an analysis of changes in serum antioxidant markers and enzyme levels. In addition, the correlation between gelatin intervention and gut microbiota was examined to gain further insights into the underlying mechanisms.

## 2. Materials and Methods

### 2.1. Materials and Reagents

The CCA and YLG powder was donated by JiXiang Biotechnology (Liaocheng, Shandong, China). The CCA and YLG are powdered and passed through a 60-mesh sieve to obtain a fine powder. The powder was stored at −20 °C until further use. The test kits for lactate dehydrogenase (LDH), lactic acid (LD), liver glycogen, muscle glycogen, Na^+^K^+^-ATPase, Ca^2+^Mg^2+^-ATPase, glucose (GLU), total antioxidant capacity (T-AOC), superoxide dismutase (SOD), blood urea nitrogen (BUN), glutathione peroxidase (GSH-Px), and malondialdehyde (MDA) were all purchased from Nanjing Jiancheng Bioengineering Institute (Nanjing, Jiangsu, China). All chemical reagents mentioned in this study were at least of analytical grade.

### 2.2. Quality Analysis

Crude protein, moisture content, mineral element, water insoluble substances and ash contents in the extracted gelatin were determined according to national standard of China [10,11,12,13] (GB/T 5009.5-2010, GB/T 5009.6-2003, GB/T 5009.268-2016, and GB/T 5009.4-2010). Color coordinates (L* (lightness), a* (redness), b* (yellowness), and ΔE (Delta E)), of samples were determined using Colorimeter (KonicaMinolta Ltd., Tokyo, Japan).

### 2.3. Scanning Electron Microscopy

Referring to Gao et al. [14], samples were crushed and placed in a vacuum drying chamber. The metal film (10 nm) was then sputtered on the surface of the sample, and the structure was magnified by 150× at a voltage of 15.0 kV.

### 2.4. Amino Acid Composition

A 100 mg sample was reacted with 2 mL HCl (6 M) at 110 °C for 24 h. Then, 100 uL of the digestion solution was blown dry with a nitrogen blower at 40 degrees and redissolved with 1 mL of water. The samples obtained after filtration were measured by high performance liquid chromatography (Shimadzu LC20AD—API 3200MD TRAP).

### 2.5. GC-MS Analysis

SPME extracted the volatile flavor components from the samples, and was performed as previously described [15]. A 3 g sample and 10 μL 2-Octanol (82.2 mg/μL) were introduced in a 20 mL glass vial. After sealing, these were equilibrated at 75 °C for 40 min. Volatile flavor substances in the headspace bottle were extracted by SPME (50/30 μm, DVB/CAR/PDMS, Supelco) with an extraction temperature of 70 °C, 50 min, desorption temperature of 250 °C and desorption time of 3 min. The extracted material were separated by VF-WAX ms column (60 m × 0.25 mm i.d. × 0.25 μm film thickness, Agilent, Santa Clara, CA, USA).

The sample was performed using a Thermo Trace 1310 GC equipped with a TriPlus RSH autosampler and ducted on a Q-Exactive Orbitrap mass analyzer. The carrier gas was Helium (99.99%), with a constant flow rate of 1.0 mL/min, the injection volume was 1 μL; the shunt ratio was 5:1; the gas inlet temperature was 250 °C. The warming temperature was programmed with a starting temperature of 40 °C, maintained for 1 min to 120 °C at 2 °C/min and then at 4 °C/min to 220 °C for 5 min. MS was performed using electron ionization (EI) circumstance (70 eV, ion source temperature 280 °C), with operation in full scan mode at a resolving power of 60,000 and scan range from 30–400 *m*/*z*.

GC–MS data were acquired and processed using the Xcalibur 4.1 and TraceFinder 4.0 software (Thermo Scientific, Bremen, Germany), respectively.

The volatile compounds were identified by comparing retention indices (RIs) and retention times with those obtained for authentic standards from NIST17 (v2.3) and IAS Center Lab. A series of alkanes (C7–C40; SigmaAldrich, St. Louis, MO, USA) were analyzed under the same chromatographic conditions to calculate LRIs. Each sample was run in triplicate.

### 2.6. Animal Experiments

Distilled water was used to dissolve the sample before it was given orally to the animals. Thirty female ICR mice (18–20 g) were purchased from Beijing Vital River Laboratory Animal Technology Co., Ltd. (Beijing, China). The mice were maintained on a 12 h/12 h day/night light cycle and provided with a standard laboratory diet and water ad libitum. The mice were randomly divided into three groups (*n* = 10), after 7-day acclimatization, which included Control (supplemented with saline solution) groups, YLG group, and CCA group at a dose of 0.2 mL, 1.2 g/kg BW/d per mouse for 4 weeks, respectively (each day at 9:30–10:00). Animal experiments were performed in accordance with Experimental Animal Welfare and Ethical of Institute of Animal Science, Chinese Academy of Agricultural Sciences guidelines (approval number: IAS2024111). During the experimental process, the body weight was recorded once weekly before gavage and observation for behavioral characteristics.

The liver and the skeletal muscle tissue of the hind legs were collected and fixed in the 10% (*w*/*v*) formalin. Feces collected after 4 weeks from each group in pairs were placed in sterile centrifuge tubes at −80 °C, resulting in five representative sample sets. Liver, kidney, heart, spleen, and skeletal muscle were removed and washed with normal saline. These were then weighed, and relative organ weight was calculated. The organ index was calculated as follows:

organ index = C_1_/C_2_, (1)

where *C*_1_ is the weight of the organ (mg), and *C*_2_ is the body weight (g).

### 2.7. Exhaustive Swimming Test

The mice from the group were used for the exhaustive swimming test as previously described [16]. Briefly, on days 25–27 of the experiment, the mice were subjected to a 10 min weight-loaded swimming training session in a swimming tank (50 × 30 × 30 cm). The swimming test was performed at a constant water temperature of 25 ± 2 °C with a fixed water depth of 25 cm. A lead block weighing 7% of the mouse’s body weight was attached to the base of the tail. After training, the mice were removed from the water, gently dried with a towel, and returned to their cages. On day 30, mice underwent the exhaustive swimming test 30 min after the last gavage. The exhaustive time was defined as the time point when mice were unable to resurface and could not reach the water surface within 7 s.

### 2.8. Analysis of Fatigue-Associated Biochemical Parameters

Blood was collected from the orbit and centrifuged at 3500 rpm for 15 min at 4 °C, and the supernatant was collected. The levels of LDH, BUN, MG, LD, Glu, CK, SOD, CAT, GSH-Px, MDA, T-AOC, Ca^2+^-Mg^2+^-ATPase, Na^+^-K^+^-ATPase, and total ATPase were measured using commercial assay kits according to the manufacturers’ protocols.

### 2.9. Hematoxylin-Eosin H&E

After 24 h of fixation, samples were dehydrated through ethanol, transparent, and embedded in paraffin and cut into 4 μm thin slices. Sections were stained with H&E and images were captured by the microscopic imaging system.

### 2.10. Fecal DNA Extraction and 16S rDNA Sequencing

DNA extraction, 16S rRNA amplification, and high-throughput sequencing were performed according to a previously described method [17]. The hypervariable region V3–V4 was amplified by primer pairs 338F (5′-ACTCCTACGGGAGGCAGCAG-3′) and 806R (5′-GGACTACHVGGGTWTCTAAT-3′).

### 2.11. Statistical Analyses

All samples were conducted a minimum of three times. Data are mean ± SD. One-way ANOVA followed by Tukey’s HSD test (SPSS 26.0) was used for pairwise comparisons among groups; *p* < 0.05 was considered significant. All figures were created using GraphPad Prism 9.4.1 (GraphPad Software INC., La Jolla, CA, USA) and the correlation heat map was drawn using ChiPlot (https://www.chiplot.online/). Multivariate statistical analyses were conducted using SIMCA^®^ 14.1 software (Umetrics, Sweden, Umeå, Sweden), with principal component analysis (PCA) performed to visualize sample clustering patterns and partial least squares-discriminant analysis (PLS-DA) implemented to compute variable importance in projection (VIP) scores for identifying discriminative biomarkers. The assessment of biochemical and histological data was performed in a blinded manner.

## 3. Results and Discussion

### 3.1. The Nutrient Component Analysis

**Figure 1:**
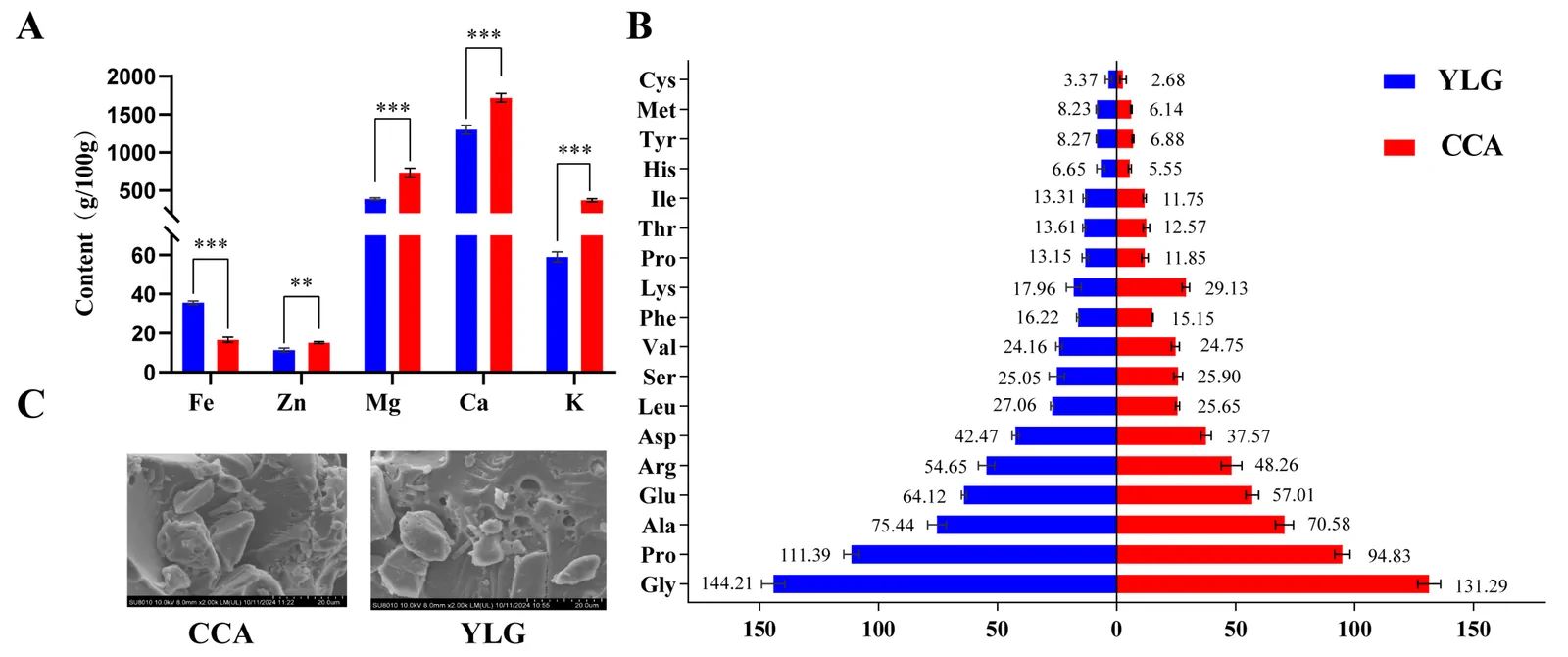
The mineral elements (**A**), amino acid content (**B**) and scanning electron micrographs (**C**) of YLG and CCA. ** and *** means *p* < 0.01 and <0.001 between groups, respectively.

The quality of randomly purchased commercial YLG complied with the requirements specified in the Pharmacopoeia. The quality parameters of YLG and CCA are summarized in Table 1. The protein content of YLG (88.19%) was found to be higher than that in CCA. The protein content is generally considered as an important indicator of quality, as it directly affects its nutritional value and effectiveness. As shown in Table 1, there are significant differences in color between YLG and CCA; these differences may be closely related to the distinct raw materials used in their production. Furthermore, the low contents of ash and water-insoluble substances are important for the quality [18]. The sample exhibited a low content of ash and water-insoluble substances (Table 1). Macroelements play an important role in maintaining human metabolic homeostasis [19]. The iron content (36.16 mg/100 g) was higher in YLG, while the contents of magnesium (776.64 mg/100 g), potassium (1446.68 mg/100 g), and calcium (375.49 mg/100 g) in CCA were higher than those in YLG (Figure 1A). Scanning electron micrographs were observed at an accelerating voltage of 20 kV (Figure 1C). As shown, YLG has a void space on the surface and a loose structure.

### 3.2. Amino Acids Composition Results

The amino acid compositions of commercially available CCA and YLG are shown in Figure 1B. Among the 18 amino acids analyzed, glycine was the most abundant amino acid in both YLG and CCA. Proline and hydroxyproline, which are major components of collagen, are important indicators for assessing the quality of gelatinous substances and play a crucial role in the stability of collagen [20]. Thermal stability is positively correlated with the content of hydroxyproline [21]. The hydroxyproline content in CCA and YLG was 11.85 mg/100 g, and 11.55 mg/100 g, respectively. Additionally, the contents of alanine and proline were relatively high. It has been reported that gelatin with higher proline and alanine content tends to show better viscoelasticity than other gelatins; however, gel strength, viscosity, rheological properties, and thermal stability were not measured in the present work, and this relationship therefore remains to be verified [21]. Acidic amino acids, such as glutamic acid (Glu) and aspartic acid (Asp), have been demonstrated to play significant roles in antioxidant activity [20], which may partly account for the antioxidant effects observed in the animal experiments, although further evidence is still needed.

### 3.3. GC-MS Results

**Figure 2:**
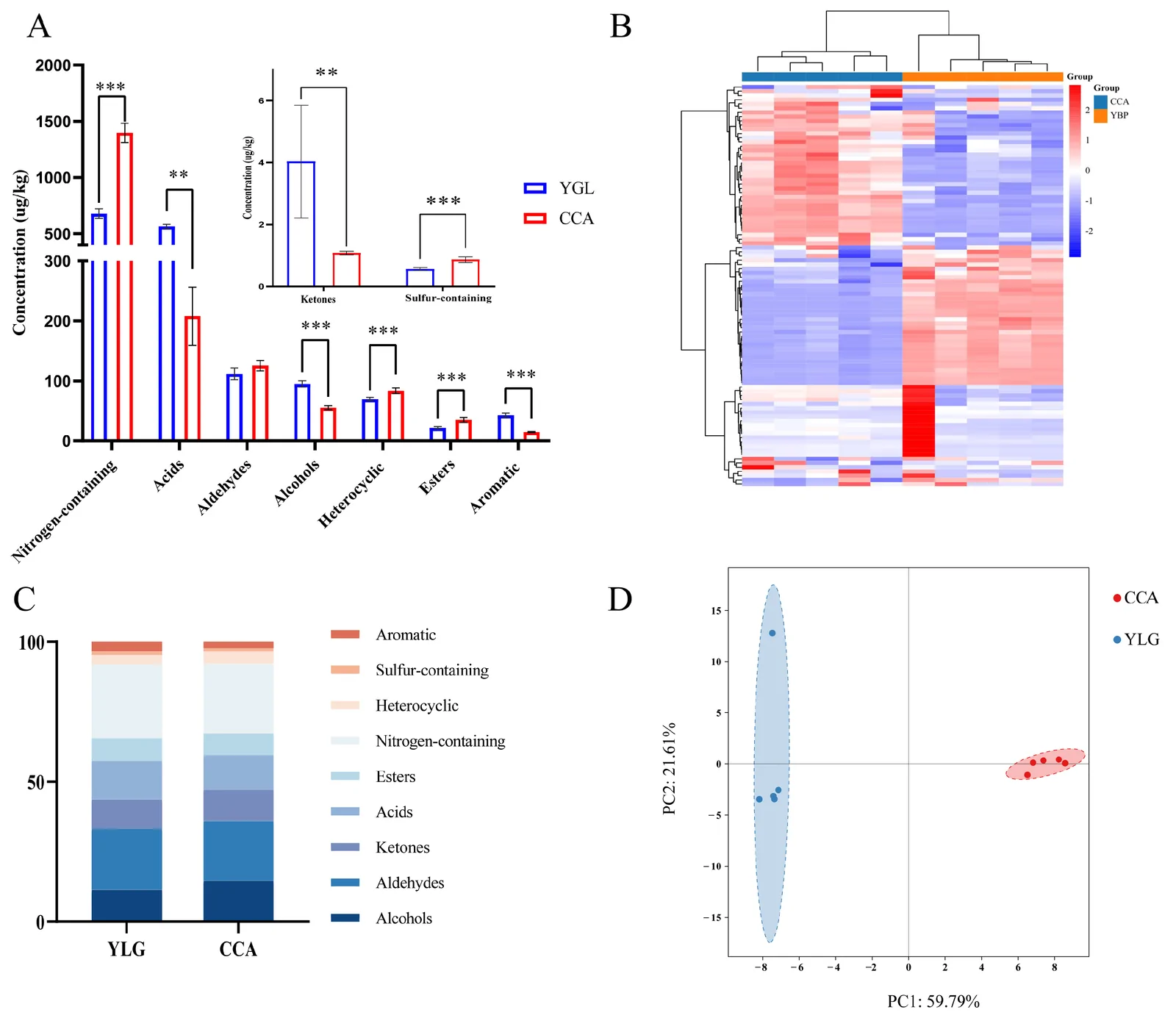
Aroma profiles with GC–MS (*n* = 5). (**A**) Categories and quantities of aroma compounds. (**B**) Heatmap of aroma compounds. (**C**) Percentages of aroma-compound categories in YLG and CCA. (**D**) PCA score chart of the GC–MS. ** *p* < 0.01; *** *p* < 0.001.

Flavor is an important factor affecting consumers’ purchasing intention. To further explore flavor characteristics, volatile flavor compounds in samples were analyzed and identified via SPME–GC-MS. GC-MS analysis of distilled samples was conducted to investigate the volatile components of Shandong-produced CCA, with 23 volatile compounds identified, 12 of which possessed characteristic odors based on OAV values and literature data (listed in Table S1) [22]. The volatile compounds identified in YLG and CCA, including the 12 characteristic odor compounds, are listed in Table S1. A total of 87 and 89 volatile compounds were detected in YLG and CCA, respectively, mainly including aldehydes, alcohols, ketones, esters, ethers, organic acids, phenols and nitrogen-containing compounds. The volatile compounds identified in the samples are listed in Figure 2A. Among all volatile flavor compounds, nitrogen-containing compounds showed the highest content in both YLG and CCA (679.82 ± 36.61 and 1397.04 ± 85.68, respectively). Because YLG and CCA are both gelatin-based products and were subjected to identical extraction, pretreatment, and GC-MS conditions, the absolute concentrations of volatile compounds were considered directly comparable. In YLG, the content of aldehydes (107.91 ± 3.24 μg/kg), alcohols (94.84 ± 4.73 μg/kg), ketones (3.36 ± 4.03 μg/kg), esters (21.74 ± 1.71 μg/kg), acids (566.46 ± 15.77 μg/kg), heterocyclic (69.43 ± 2.91 μg/kg), aromatic (42.67 ± 3.05 μg/kg), sulfur-containing (0.56 ± 0.04 μg/kg) and nitrogen-containing compounds (679.82 ± 36.61 μg/kg) were all significantly higher than those in CCA (Figure 2C, *p* < 0.05).

Among the identified volatiles, nitrogen-containing compounds are the most abundant chemical group in YLG and CCA. A total of 23 and 22 nitrogen-containing compounds were detected in YLG and CCA, respectively. Pyrazines were the predominant nitrogen-containing compounds in both samples, mainly presenting roasted nut and roasting aroma characteristics. Pyrazine derivatives emerged as the dominant subclass, predominantly featuring 2,3-dimethylpyrazine, trimethylpyrazine, 2-ethyl-5-methylpyrazine, and 2,6-diethylpyrazine. Based on OAV values and literature data, these four compounds were considered to synergistically contribute to a unique roasted, nutty and smoky aroma in YLG, which was inferred to show higher olfactory complexity than the simple 2,5-dimethylpyrazine-dominated aroma of CCA. Mechanistically, pyrazines are heterocyclic nitrogen-containing compounds formed predominantly through the Maillard reaction and Strecker degradation of amino acids during thermal processing [22]. The structure of the pyrazine ring, particularly the number and position of alkyl or acetyl substituents, strongly influences both odor character and sensory threshold: alkylpyrazines generally impart nutty and roasted notes, whereas acetylpyrazine exhibits a popcorn-like, bread-crust aroma with an extremely low odor threshold.

To further screen the differential volatile components between YLG and CCA, variable importance in the projection (VIP) derived from the PLS-DA model was applied. A VIP value greater than 1 is recognized as a valid threshold indicating that the corresponding variable contributes significantly to sample differentiation [22,23]. Volatile flavor compounds with VIP > 1 and *p* < 0.05 were further selected as the key differential aroma compounds of gelatin products, and a total of 45 volatile compounds were screened out accordingly (Table S2). On this basis, the odor activity value (OAV) was calculated to evaluate the actual sensory contribution of these screened volatiles.

The effects of volatile flavor compounds on sensory characteristics depend not only on their concentrations but also on their olfactory thresholds [24,25]. Only volatile compounds with sufficient odor activity values (OAVs) can be perceived by sensory evaluation. The odor activity value, defined as the ratio of a compound’s absolute concentration to its detection threshold, serves as a critical determinant: compounds with OAV > 1 are considered key flavor contributors, while those exceeding OAV > 100 play a dominant role in shaping the characteristic flavor profile [24]. YLG contained nine compounds with OAVs exceeding 100, including 2,3-octanedione, phenethyl alcohol, *p*-cresol, acetylpyrazine, pentanal, 3,5-diethyl-2-methylpyrazine, nonanal, hexanal and 3-methylbutanal. In contrast, CCA exhibits ten such high-OAV compounds: methylpyrazine, 3-phenylpropenal, ethylpyrazine, 2,3-dimethylpyrazine, *p-cresol*, acetylpyrazine, pentanal, nonanal, hexanal, and 3-methylbutanal. Among the nine high-OAV compounds in YLG, 3,5-diethyl-2-methylpyrazine, acetylpyrazine and 3-methylbutanal were mainly associated with roasted, nutty and popcorn-like notes; *p-cresol* contributed a smoky, phenolic character; pentanal, hexanal and nonanal imparted fruity, green and fatty nuances; and 2,3-octanedione and phenethyl alcohol provided buttery, fruity and floral notes. The elevated OAVs of 2,3-octanedione and phenethyl alcohol suggest their potential contribution to fruity and floral nuances, which may confer enhanced aromatic complexity to YLG compared to CCA. Notably, 3-phenylpropenal and 2,3-dimethylpyrazine in CCA were likely associated with nutty-roasted attributes. Aldehydes including pentanal, hexanal, nonanal and 3-methylbutanal showed strong odor potency in both samples. Their supra-threshold concentrations (OAV > 100) made them the main odor compounds responsible for fruity and nutty flavors.

To further explore the aroma differences among samples, 92 volatile compounds were selected as dependent variables, and sample groups were set as independent variables. PCA was employed to distinguish the three samples. Figure 2D shows the scatter plot of the PCA model scores for the different samples. The results revealed significant differences in aroma compounds between the two samples, which was further verified by heatmap analysis (Figure 2B). Notably, compared with the 2,5-dimethylpyrazine-dominated profile of CCA [22], the diversified alkylpyrazine profile of YLG reflects its distinct processing conditions and amino acid composition, supporting the uniqueness of its roasted, nutty and smoky aroma.

### 3.4. The Anti-Fatigue Capacity

During the experiment, the body weight of mice in all groups increased steadily, with no significant differences among groups over the feeding period. Moreover, no abnormalities in diet or physical condition were observed. Additionally, no significant differences were found in organ indices, indicating that YLG and CCA supplementation did not adversely affect normal growth of mice under the present experimental conditions (Table 2). Exercise capacity is one of the most direct methods to demonstrate anti-fatigue effects [26].

**Figure 3:**
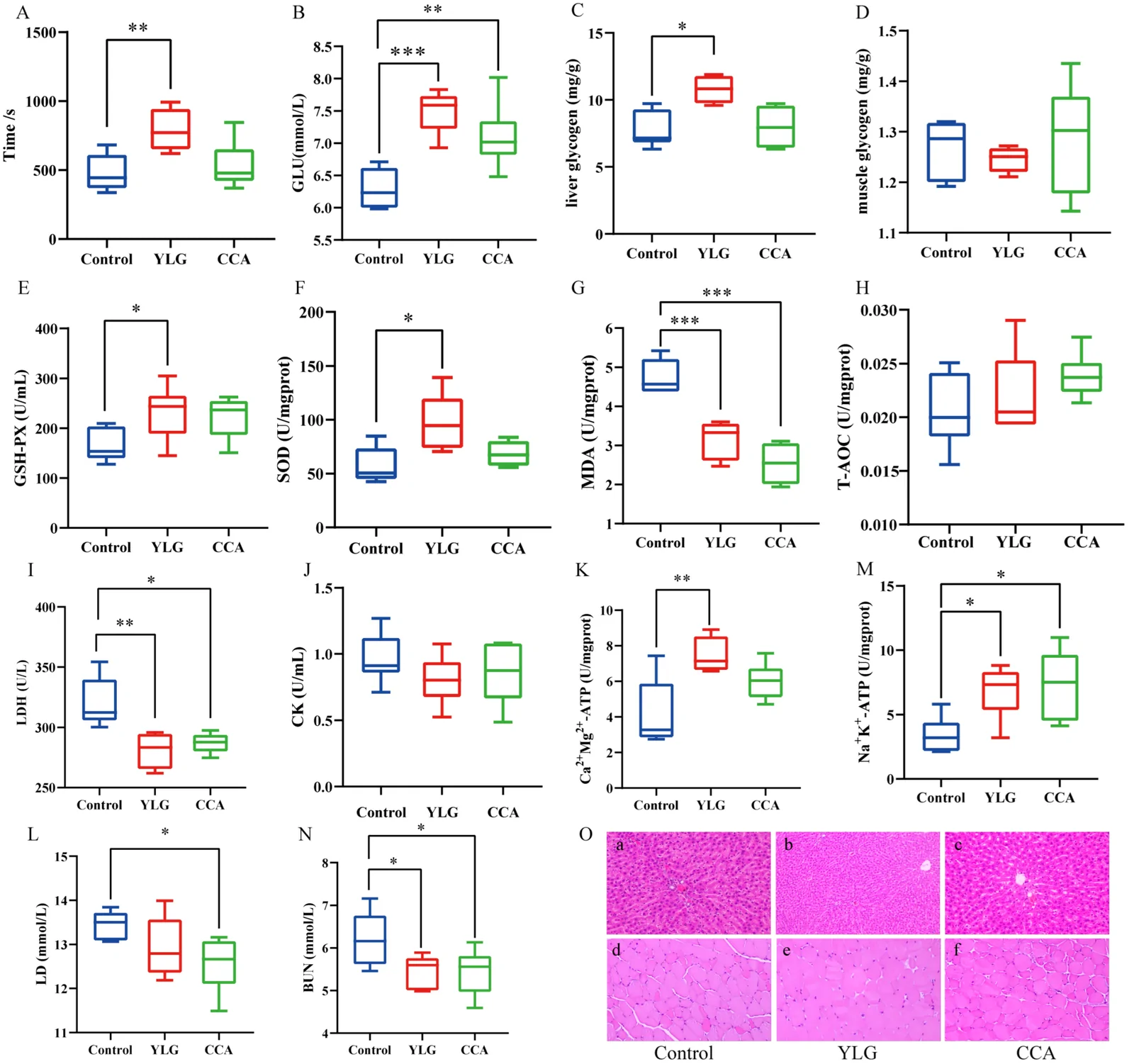
The swimming time of mice after weight bearing (**A**). The content of glucose (Glu), liver glycogen, muscle glycogen, glutathione peroxidase (GSH-PX), superoxide dismutase (SOD), malondialdehyde (MDA), total antioxidant capacity (T-AOC), lactic dehydrogenase (LDH), creatine kinase (CK), Ca^2+^-Mg^2+^-ATPase, Na^+^-K^+^-ATPase, lactic acid (LD) and blood urea nitrogen (BUN), in the blood of mice after forced swimming (**B**–**N**). The hematoxylin–eosin (HE) staining of the mice liver and skeletal muscle (**O**) ((**Oa**–**Oc**) liver cells, (**Od**–**Of**) skeletal muscle). * *p* < 0.05; ** *p* < 0.01; *** *p* < 0.001. Data are expressed as mean ± SD.

The weighted swimming test is the most commonly used animal model for assessing fatigue levels. The time to exhaustion in the weighted swimming test is a direct indicator of exercise endurance [27]. Mice often exhibit initial discomfort during the first swimming session, which may result in shorter swimming durations; however, no significant differences were observed among groups at that stage. Therefore, three swimming training sessions were conducted prior to the exhaustive swimming test to minimize experimental error. After the final administration, the exhaustive swimming test was conducted to evaluate the effects of YLG and CCA on the exercise capacity of mice. The exhaustive swimming time of mice in both the YLG group and the CCA group increased, showing an increase of 64% (*p* < 0.05) and 11%, respectively, compared to the control group (Figure 3A). These results suggested that the treatments could enhance the exercise endurance of mice and prolong their time to exhaustion in the swimming test.

### 3.5. Energy Substances and HE

Glycogen is an important energy source during exercise, as it helps maintain blood glucose levels [28]. Energy is provided through the rapid conversion of glycogen into ATP (adenosine triphosphate), which offers efficient energy for muscles and supports prolonged exercise through glycogen reserves in the liver and muscle. Adequate liver and muscle glycogen reserves can significantly enhance intensive exercise performance, delay fatigue, and help prolong exercise time [26]. As shown in Figure 3B, the Glu content in the intervention group was significantly higher than that in the control group (*p* < 0.05), increasing by 19.30% and 12.96%, respectively. As shown in Figure 3C, hepatic glycogen content after exhaustive exercise in the treatment groups was significantly higher than that in the control group (*p* < 0.05). All remaining experimental groups exhibited numerically elevated glycogen levels relative to controls, although none of these differences reached statistical significance. Compared with the control group, muscular glycogen levels in the sample groups increased without significant difference (*p* > 0.05). This may be attributed to rapid muscle glycogen depletion induced by acute fatigue in the exercised mice, as the short rest period was insufficient to restore this index to normal levels.

Excessive exercise can generally have adverse effects on the body’s organs and tissues. Studies have demonstrated that exhaustive exercise can damage hepatocytes by altering hepatic blood flow and increase the risk of muscle injury, thereby destroying skeletal muscle integrity and impairing normal muscle cell function [29,30]. As the main metabolic organ, the liver is responsible for detoxification, and changes in its histological morphology are an important index for toxicological evaluation [30]. As shown in Figure 3(Oa–Oc), hepatocyte arrangement, structure and size exhibited no obvious differences relative to the control group, indicating that no obvious acute liver injury occurred in the mice under the present experimental conditions, but further toxicological studies are still needed for overall safety assessment. Exhaustive exercise can induce skeletal muscle injury, disrupt tissue integrity and impair normal myocyte function. As shown in Figure 3(Od–Of), skeletal muscle damage was markedly alleviated in the YLG and CCA groups compared with the control group; these groups showed clearer and more uniform staining as well as distinct nuclei. Moreover, muscle cells in the YLG group were more tightly arranged, indicating that YLG contributed more effectively to skeletal muscle recovery.

### 3.6. Effects on Oxidative Stress and Exercise-Induced Muscle Damage

Intense exercise consumes large amounts of oxygen, promoting the generation of free radicals and reactive oxygen species (ROS) [26]. These by-products accelerate lipid peroxidation, tissue damage, and muscle strength reduction, leading to fatigue. Studies have shown that excessive free radical production after intense exercise disrupts the balance between oxidative and antioxidant systems, causing lipid peroxidation and impairing energy synthesis [18,31]. Chronic oxidative stress can lead to the accumulation of free radicals and MDA, damaging tissues and organs and exacerbating fatigue [32]. The enzymatic antioxidant system (such as SOD and GSH-Px) helps eliminate free radicals and maintain cellular function [16,33]. This study examined the effects of YLG and CCA on GSH-Px and SOD activities in exhausted mice, and MDA and T-AOC levels were measured to assess oxidative stress.

MDA is a reactive oxygen species generated via enzymatic and non-enzymatic pathways in vivo. It attacks polyunsaturated fatty acids in cell membranes and triggers lipid peroxidation and lipid peroxide formation [30]. Intense exercise leads to excessive production of oxygen free radicals, further promoting lipid peroxidation and MDA formation, which disrupts biological membranes and mitochondrial function, ultimately resulting in fatigue. T-AOC level is widely used to evaluate the overall antioxidant capacity in vivo. T-AOC helps alleviate exercise-induced fatigue and enhance the body’s anti-fatigue capacity by scavenging free radicals, reducing oxidative damage, and protecting cellular function [34]. Jiang et al. [35] reported that two polysaccharides, BCP-1 and BCP-2, obtained from *Bupleurum chinense* DC, improved the antioxidant system following exercise.

The obtained results are depicted in Figure 3E,F. After exercise, the levels of SOD and GSH-Px significantly increased, especially in the YLG group (*p* < 0.05). Although the T-AOC content was higher compared to the control group, the difference was not significant (Figure 3G, *p* > 0.05). The MDA concentration significantly decreased after sample intervention (Figure 3H, *p* < 0.05), with reductions of 33.51% and 47.94%, respectively, indicating that YLG and CCA could effectively inhibit lipid peroxidation in mice. Comprehensive analysis indicated that YLG supplementation could effectively increase SOD and GSH-Px activities and reduce MDA levels. By maintaining the balance between the oxidative and antioxidative systems, it protects cells from oxidative stress damage and alleviates exercise-induced fatigue.

In addition, the protective effect of YLG against exercise-induced muscle damage was also evaluated. CK and LDH are key biomarkers of tissue damage during high-intensity exercise [36]. The levels of LDH and CK were found to be significantly reduced in both the YLG and CCA groups (Figure 3G–I, *p* < 0.05), suggesting that YLG and CCA may reduce exercise-induced muscle membrane damage. Excessive ROS produced during exhaustive exercise can disrupt the integrity of skeletal muscle cell membranes, leading to the leakage of intracellular enzymes such as CK and LDH into the bloodstream [26]. Lower serum CK and LDH levels in YLG-treated mice indicate less oxidative damage to muscle cell membranes, but this conclusion is based on biochemical markers alone.

### 3.7. ATPase Activity and Fatigue-Related Metabolites

The activity of Na^+^-K^+^-ATPase and Ca^2+^-Mg^2+^-ATPase enzymes during exercise is highly dependent on intracellular ATP levels. As exercise progresses, ATP levels gradually decline, particularly when glycogen stores in the muscles are depleted [37]. This reduction in ATP availability leads to a decreased rate of ATP synthesis, resulting in the functional impairment of both Na^+^-K^+^-ATPase and Ca^2+^-Mg^2+^-ATPase enzymes. These changes disrupt energy metabolism, contributing to muscle fatigue and further diminishing exercise capacity [38]. As shown in Figure 3K, Ca^2+^-Mg^2+^-ATPase activity was significantly higher in the YLG group (*p* < 0.05). The CCA group showed an increasing trend without significant difference compared with the control group (*p* > 0.05). As shown in Figure 3M, Na^+^-K^+^-ATPase activity in all intervention groups was significantly higher than that in the control group (*p* < 0.05). YLG and CCA significantly reduced BUN and LD levels (*p* < 0.05), indicating a lower accumulation of fatigue-related metabolites. These changes may reflect an effect on energy metabolism.

LD and BUN levels are indicators of energy expenditure and metabolic stress [26]. During physical activity, the excessive buildup of metabolites such as lactate, phosphates, and urea nitrogen can disrupt normal physiological functions, contributing to fatigue [39,40,41]. LD is a metabolic product generated through the reduction of pyruvate during glycolysis [41]. Changes in LD concentration can serve as indicators of energy expenditure and metabolic stress during intense exercise or under varying physiological loads [33]. As shown in Figure 3L, LD levels in both YLG and CCA groups decreased compared with the control group, with a reduction of 3.64% and 6.45%, respectively; the reduction in the CCA group was significant (*p* < 0.05). BUN, a key metabolite of protein catabolism, plays a crucial role in reflecting both liver protein synthesis and kidney excretion functions. Its levels are not only positively correlated with exercise tolerance but also serve as an important indicator of metabolic stress. During intense physical activity, the body undergoes increased protein catabolism to compensate for energy demands, leading to elevated BUN concentrations [42,43]. As shown in Figure 3N, the values in both the YLG and CCA gastric administration groups were lower than those in the control group, with reductions of 12.21% and 12.28%, respectively (*p* < 0.05). The results confirmed that YLG and CCA interventions significantly reduced BUN and LD levels (*p* < 0.05) and effectively restored fatigue-related biomarkers to normal levels.

### 3.8. Gut Microbiome

**Figure 4:**
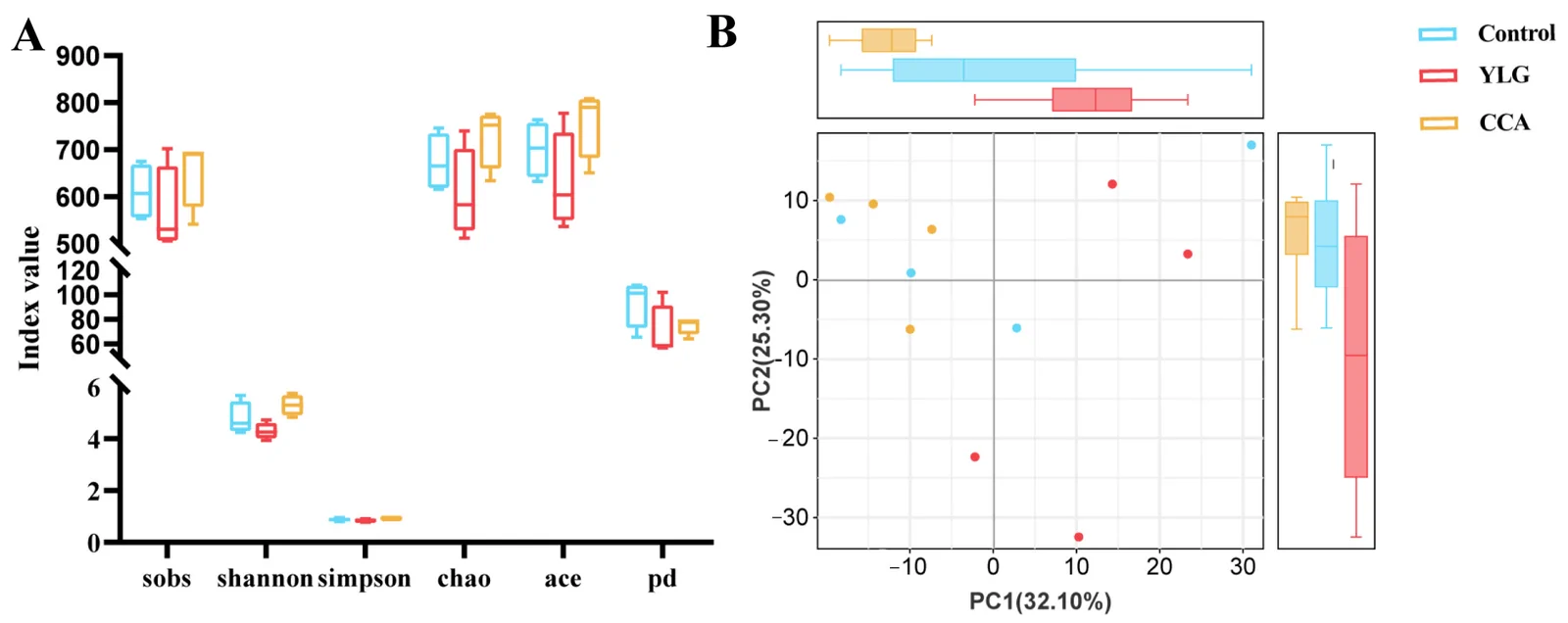
The α-diversity (**A**) and β-diversity (**B**) analysis of gut microbiota in different groups.

Recent studies suggest that gut microbiota play a key role in regulating fatigue. Studies in mouse models indicate that a healthy microbiome may improve physical endurance and recovery [8,44]. Therefore, the 16s rRNA analysis was performed on fecal samples. The results are shown in Figure 4A. The α-diversity indices, including Sobs, Shannon, Simpson, Chao, Ace, and phylogenetic diversity (pd), showed no significant differences when compared to the control group (*p* > 0.05). This indicates that the samples did not affect the richness and diversity of gut microbiota. Principal component analysis (PCA) results, based on β-diversity analysis, indicated no significant difference in community structure diversity among the three groups (Figure 4B).

**Figure 5:**
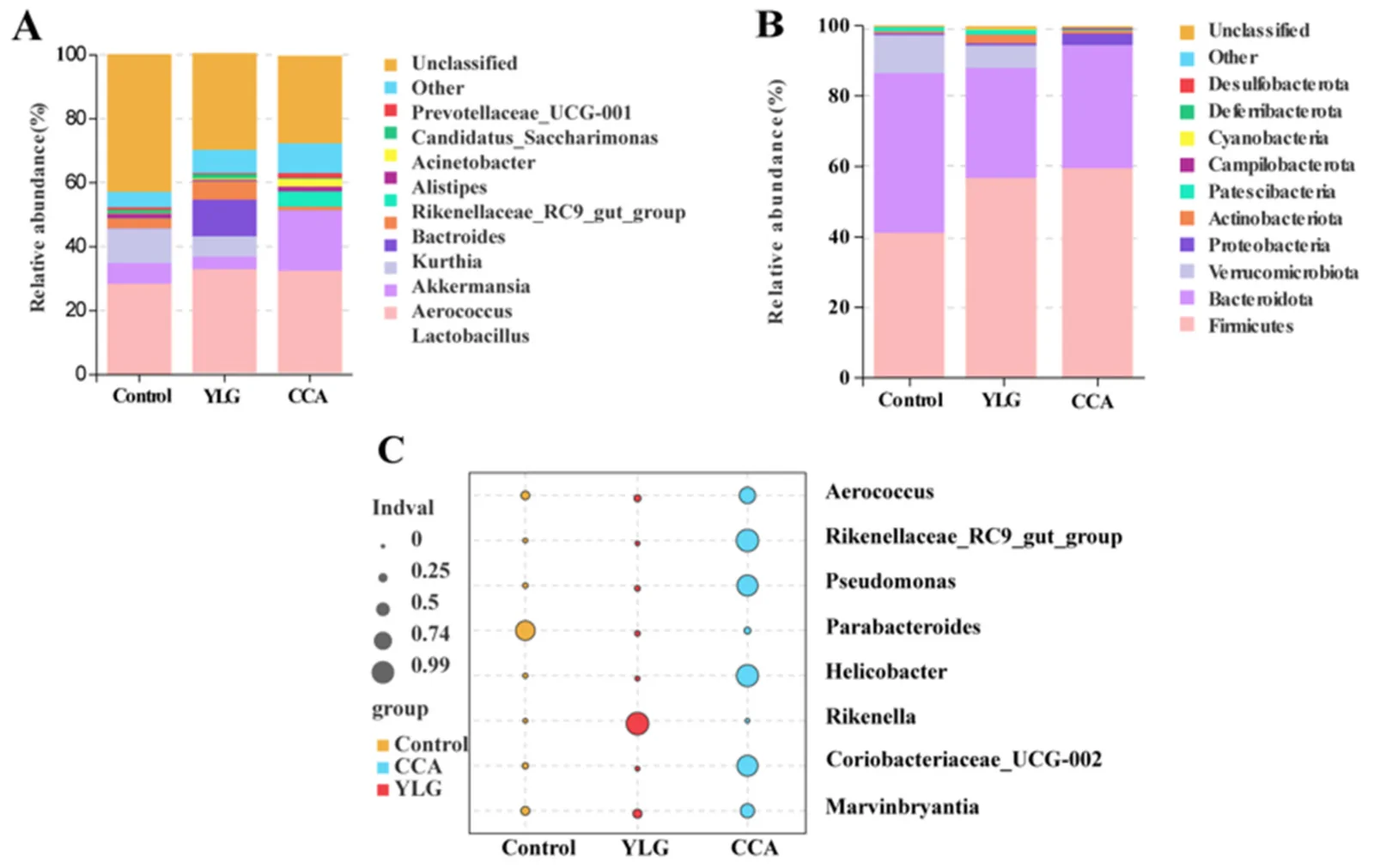
The fecal microbiome composition at the phylum level (**A**) and genus level (**B**). Signature bacterial genera of the Control, CCA and YLG groups are shown by indicator species analysis (**C**). Each group had four parallels of mixed samples derived from ten mice, represented in the results from these parallel samples.

These results indicate that the treatments did not cause a global shift in the gut microbial community. At the taxonomic level, however, the relative abundance of specific bacterial taxa changed significantly. Dietary supplementation with CCA and YLG increased the relative abundance of *Firmicutes*, *Proteobacteria*, *Actinobacteriota* and *Cyanobacteria*, while the abundances of *Bacteroidota*, *Verrucomicrobiota*, and *Deferribacterota* decreased (Figure 5A,B). At the genus level, CCA and YLG supplementation increased the abundance of *Lactobacillus* and *Acinetobacter*, while the abundances of *Aerococcus* and *Akkermansia* decreased. *Lactobacillus* is a predominant gut bacterium in endurance athletes and plays a key role as a probiotic by enhancing protein utilization, boosting branched-chain amino acid levels, supporting muscle energy balance, and delaying fatigue onset [45]. In the YLG group, *Kurthia* and *Bacteroides* were up-regulated, while *Rikenellaceae RC9* gut group and *Alistipes* showed down-regulation. The strain *Bacteroides* is connected with muscle mass and function [46]. Poor cognition is associated with an increased abundance of *Alistipes*. The *Alistipes* genus, which is linked to anxiety, chronic fatigue syndrome, depression, and hypertension, was found to be more abundant in the group with poor cognitive function [47]. Indicator species analysis identified signature bacterial genera characteristic of the YLG, CCA and control groups (Figure 5C). LEfSe analysis further confirmed bacterial taxa with statistically significant intergroup differences, and the complete results are provided in Figure S1. These results suggest that the anti-fatigue effects of YLG and CCA are associated with changes in specific intestinal bacterial taxa, and that YLG had a more pronounced effect on these taxa. In this study, only the composition of the gut microbiota was analyzed, and related metabolites such as short-chain fatty acids and amino acids were not measured. Further metabolomic studies are needed to clarify how these bacterial changes may contribute to the anti-fatigue effects.

## 4. Conclusions

The present study demonstrates that YLG derived from yak by-products exhibits distinct nutritional characteristics compared with traditional CCA, including a higher protein content. As auxiliary basic attributes, YLG presents unique flavor profiles predominantly characterized by pyrazine-related nutty and smoky aromas, which are obviously different from those of CCA. The core focus of this work lies in the anti-fatigue performance and potential mechanisms of YLG. In vivo anti-fatigue experiments demonstrated that YLG supplementation improved exercise endurance and enhanced antioxidant capacity, and alleviated exercise-induced skeletal muscle damage, as reflected by the improved biochemical parameters associated with energy metabolism. Furthermore, YLG modulated the abundance of specific intestinal bacterial taxa associated with fatigue alleviation. This finding suggests a potential link between gut microbiota and the observed anti-fatigue effects. The present results support our hypothesis that YLG exerts stronger anti-fatigue effects than CCA, as evidenced by the significantly prolonged exhaustive swimming time and improved biochemical markers in the YLG group. Overall, the anti-fatigue effects of YLG were accompanied by changes in energy metabolism-related parameters, antioxidant defense, muscle damage markers, and specific gut bacterial taxa. The molecular mechanisms behind these effects remain to be determined. This study supports the high-value utilization of yak skin by-products as a promising natural raw material for developing anti-fatigue functional foods.

## Figures and Tables

**Table 1:** YLG and CCA nutrient component.

	YLG	CCA	*p*
Moisture content %	10.15 ± 0.06	9.87 ± 0.25	-
Water-insoluble substances %	1.57 ± 0.12	0.73 ± 0.05	***
Ash contents %	0.76 ± 0.05	1.36 ± 0.11	**
Crude protein %	88.19 ± 1.86	82.02 ± 1.78	*
L*	27.75 ± 0.24	25.45 ± 0.24	***
a*	4.79 ± 0.08	3.82 ± 0.05	***
b*	5.28 ± 0.45	5.39 ± 0.16	-
ΔE	66.21 ± 0.27	68.51 ± 0.27	***

* *p* < 0.05; ** *p* < 0.01; *** *p* < 0.001. “-”: no significant difference.

**Table 2:** Effect on organ index in mice.

Group	Cardiac Index/%	Liver Index/%	Spleen Index/%	Renal Index/%
Control	0.54 ± 0.05	5.62 ± 0.70	0.53 ± 0.15	1.23 ± 0.13
YLG	0.57 ± 0.05	5.46 ± 0.31	0.48 ± 0.10	1.37 ± 0.23
CCA	0.61 ± 0.13	5.41 ± 0.76	0.46 ± 0.1	1.27 ± 0.17

## Data Availability

The original contributions presented in this study are included in the article/Supplementary Materials. Further inquiries can be directed to the corresponding author.

## Supplementary Materials

The following supporting information can be downloaded at: https://www.mdpi.com/article/10.3390/foods15203623/s1, Table S1: Volatile compounds identified in YLG and CCA samples; Table S2: YLG and CCA are the key volatile flavor substances; Figure S1: The model microbiomes were identified using LEfSe analysis. Each group had four parallels of mixed samples derived from ten mice represented the results from these parallel samples.

## Abbreviations

The following abbreviations are used in this manuscript:

CCA	Colla Corii Asini
YLG	Yak Leather Gel
SOD	superoxide dismutase
LDH	lactate dehydrogenase
LD	lactic acid
GLU	glucose
T-AOC	total antioxidant capacity
BUN	blood urea nitrogen
GSH-Px	glutathione peroxidase
MDA	malondialdehyde
