# Supplementary material for: Quality Characterization and Anti-Fatigue Properties of Yak (Bos grunniens) Leather Gel in a Mouse Endurance Swimming Model
Source: Foods. 2026 Oct 12;15(20):3623. doi: 10.3390/foods15203623 (PMC13703379; doi:10.3390/foods15203623)
Supplement: Supplementary file 1 [file foods-15-03623-s001.zip › foods-4542290-supplementary.pdf]

Supplementary Table S1

Table S1 Volatile compounds identified in YLG and CCA samples

No.	Component Name	CAS No.	Avg Calculated RI	Library RI	Concentration (μg/kg)	
					YLG	CCA
1	(Z)-5-OCTEN-1-OL	64275-73-6	1599	1615	1.44±0.24	——
2	1-Butanol	71-36-3	1130	1150	1.5±0.12	——
3	1-Decanol	112-30-1	1746	1760	0.36±0.06	0.49±0.08
4	1-Dodecanol	112-53-8	1950	1969	0.73±0.13	0.28±0.06
5	1-Pentanol	71-41-0	1236	1253	11.64±0.58	——
6	1-OCTEN-3-OL	53907-72-5	1435	1451	1.13±0.08	——
7	2-Propyl-1-pentanol	58175-57-8	1438	1485	2.8±0.5	2.32±3.26
8	2-Octanone	111-13-7	1279	1293	5.95±2.65	1.42±0.2
9	1-Hexanol, 2-ethyl-	104-76-7	1475	1491	1.7±0.19	6.48±1.11
10	3-Octanol, 3,7-dimethyl-	78-69-3	1419	1400	0.95±0.19	2.42±0.1
11	Benzenemethanol	100-51-6	1861	1880	6.41±0.38	0.62±0.06
12	Benzeneethanol	60-12-8	1897	1926	17.68±2.06	7.1±0.32
13	Citronellyl acetate	150-84-5	1629	1667	2.42±0.21	2.3±0.13
14	Cedrol	77-53-2	2119	2116	3.15±0.54	0.89±0.21
15	2-Decenal, (E)-	3913-81-3	1640	1644	0.39±0.08	0.18±0.06
16	2-Nonenal, (E)-	18829-56-6	1532	1547	1.9±0.22	2.49±1.93
17	(E)-2-Undecenal	53448-07-0	1747	1752	0.24±0.06	——
18	2-Pentenal, (E)-	1576-87-0	1124	1138	——	0.83±0.57
19	2,4-HEPTADIENAL ,(E,E)-	4313-03-5	1490	1507	1.46±0.2	0.78±0.27
20	(Z)-3-Phenylacrylald ehyde	57194-69-1	1863	1884	0.64±0.16	0.51±0.06
21	Benzaldehyde, 2-hydroxy-	90-02-8	1677	1672	0.47±0	0.44±0.1
22	2-Thiophenecarboxal dehyde	98-03-3	1688	1684	1.15±0.02	1.32±0.06
23	2-Octenal	2363-89-5	1426	1440	2.71±0.29	1.88±0.1
24	2-Propenal, 3-phenyl-	104-55-2	2037	2005	3.79±0.5	0.98±0.1
25	Butanal, 3-methyl-	590-86-3	910	923	0.34±0.11	0.61±0.05

26	Benzaldehyde, 4-methoxy-	123-11-5	2022	2011	2.96±0.38	1.92±0.13
27	Benzaldehyde	100-52-7	1520	1541	45.5±1.48	52.3±2.88
28	Benzeneacetaldehyd e	122-78-1	1639	1661	1.86±0.18	1.81±0.08
29	Heptanal	111-71-7	1180	1182	2.46±0.31	3.82±2.28
30	Hexanal	66-25-1	1075	1077	21.16±2.99	11.58±1.06
31	Nonanal	124-19-6	1390	1402	30.99±2.81	24.24±1.12
32	TRANS,TRANS-NO NA-2,4-DIENAL	5910-87-2	1697	1713	1.73±0.28	0.88±0.07
33	Pentanal	110-62-3	971	969	3.54±0.13	1.4±0.08
34	Octanal	124-13-0	1285	1298	2.22±0.53	0.21±0.01
35	1-Octen-3-one	4312-99-6	1295	1300	0.23±0.02	0.21±0.01
36	2,3-Octanedione	585-25-1	1316	1323	0.29±0.03	2.37±0.16
37	2-Heptanone	110-43-0	1176	1190	2.62±0.23	1.25±0.12
38	2-Nonanone	821-55-6	1384	1390	0.25±0.02	0.48±0.04
39	3-Octen-2-one	1669-44-9	1402	1417	2.1±0.14	1.89±0.92
40	2-Hexanone, 5-methyl-	110-93-0	1330	1346	2.78±0.31	1.9±0.26
41	6-Methyl-5-hepten-2 -one	119-61-9	2477	2450	0.41±0.07	0.29±0.05
42	Benzophenone	123-35-3	1115	1161	0.76±0.06	0.45±0.11
43	HYDROXY DIMETHYL FURANONE	14901-07-6	1934	1971	0.28±0.04	20.33±1.41
44	.beta.-Myrcene	116-53-0	1653	1662	6.28±0.52	45.48±3.57
45	.beta.-Ionone	64-18-6	1504	1503	——	0.63±0.04
46	Butanoic acid, 2-methyl-	646-07-1	1785	1803	2.13±0.27	21.73±1.32
47	Pentanoic acid, 4-methyl-	65-85-0	2418	2412	3.3±0.64	3.23±0.3
48	Benzoic acid	103-82-2	2538	2565	0.65±0.22	5.79±0.53
49	Benzeneacetic acid	79-09-4	1523	1561	12.03±0.55	105.02±5.63
50	Propanoic acid	107-92-6	1610	1649	34.32±2.17	178.75±12.32
51	Butanoic acid	111-14-8	1933	1970	4.52±0.53	4.69±2.43
52	Heptanoic acid	334-48-5	2252	2289	5.15±0.75	14.45±1.63

53	Decanoic acid	142-62-1	1826	1864	86.42±6.93	79.87±27.41
54	Hexanoic acid	501-52-0	2604	2637	0.23±0.1	2.31±0.27
55	Hydrocinnamic acid	109-52-4	1719	1733	22.31±1.84	90.26±4.5
56	Pentanoic acid	124-07-2	2040	2077	30.54±42.05	14.25±5.09
57	Octanoic acid	4312-99-6	1295	1300	0.49±0.07	1.21±0.23
58	.gamma. DECALACTONE	706-14-9	2140	2138	4.79±0.18	3.92±0.61
59	Butyrolactone	96-48-0	1625	1652	3.7±0.4	4.09±0.15
60	Hexanedioic acid, dimethyl ester	627-93-0	1803	1807	3.11±0.38	2.04±0.26
61	Formic acid, octyl ester	112-32-3	1542	1553	20.04±2.99	7.6±1.2
62	Dimethyl phthalate	131-11-3	2285	2303	0.64±0.04	0.58±0.05
63	Dodecanoic acid, methyl ester	111-82-0	1794	1804	0.9±0.08	1.66±0.06
64	Pentanedioic acid, dimethyl ester	1119-40-0	1687	1687	772.74±51.08	234.7±15.09
65	Hexadecanoic acid, methyl ester	112-39-0	2207	2208	0.64±0.08	2.92±0.32
66	Pyrazine, 2-methyl-5-(1-propenyl)-, (Z)-	55138-66-4	1533	1535	85.79±4.62	133.89±5.86
67	Pyrazine, 2,3-dimethyl-	5910-89-4	1315	1325	0.82±0.05	1.76±0.09
68	2,5-Dimethyl-3-n-pentylpyrazine	56617-69-7	1699	1680	——	0.76±0.04
69	Pyrazine, 2,6-diethyl-	13067-27-1	1439	1432	102.58±6.09	63.44±3.38
70	Pyrazine, 2-methyl-3-(2-propenyl)-	55138-62-0	1709	1739	3.3±0.3	8.42±0.48
71	2-Methyl-3-propylpyrazine	15986-80-8	1458	1462	0.32±0.02	1.13±0.05
72	Pyrazine, 2-ethyl-5-methyl-	13360-64-0	1384	1382	6.12±0.41	18.04±0.53
73	Pyrazine, 2-ethenyl-6-methyl-	13925-09-2	1483	1490	0.36±0.02	0.77±0.03
74	3,5-Dimethyl-2-isobutylpyrazine	70303-42-3	1541	1549	0.37±0.03	1.27±0.05
75	Pyrazine, 3,5-diethyl-2-methyl	18138-05-1	1487	1496	219.92±13.05	59.4±3.27

	-					
76	5H-5-Methyl-6,7-dihydrocyclopentapyrazine	23747-48-0	1616	1627	176.27±9.46	137.85±7.68
77	5H-Cyclopentapyrazine, 6,7-dihydro-2,5-dimethyl-	38917-61-2	1669	1672	25.64±1.35	11.62±0.69
78	Pyrazine, methyl-	109-08-0	1258	1263	1.28±0.11	2.19±0.33
79	Pyrazine, trimethyl-	14667-55-1	1396	1402	0.86±0.09	0.71±0.07
81	Pyrazine, ethyl-	13925-00-3	1327	1333	0.45±0.17	1.09±0.61
82	acetylpyrazine	N/A	1621	1642	2.26±0.12	1.91±1.99
83	Disulfide, dimethyl	624-92-0	1066	1071	0.57±0.06	0.51±0.11
84	Phenol	108-95-2	1988	2024	0.51±0.08	0.42±0.56
85	Eugenol	97-53-0	2152	2169	0.15±0.12	8.15±0.52
86	Phenol, 2,3,6-trimethyl-	2416-94-6	1986	2031	4.94±0.35	2.85±0.11
87	Pyridine, 2,4,6-trimethyl-	108-75-8	1363	1371	5.18±0.34	1.91±0.1
88	Pyridine, 2-methyl-	109-06-8	1209	1219	1.1±0.07	1.53±0.09
89	Pyridine, 2-pentyl-	2294-76-0	1568	1578	0.56±0.06	——
90	Pyridine, 2-ethyl-	100-71-0	1275	1278	0.75±0.02	50.35±1.26
91	Pyridine, 3-ethyl-	536-78-7	1376	1377	67.05±3.59	0.56±0.04
92	2-Acetyl-3-methylthiophene	13679-72-6	1760	1761	10.09±0.51	8.4±0.6
93	2-Acetyl-5-methylthiophene	13679-74-8	1865	1878	——	2.52±0.1
94	Thiophene	110-02-1	1016	1031	4.8±0.48	31.76±2.44

Supplementary Table S2

Table S2 YLG and CCA are the key volatile flavor substances

NO.	Compound name	VIP	*p*	Odor threshold (mg/kg)	OAV	
					YLG	CCA
1	β-Ionone	1.49	0	0.5	40.66	2.66
2	3,5-Dimethyl-2-isobutylpyrazine	1.49	0	—	—	—
3	Methylpyrazine	1.47	0	0.6	99.00	366.54
4	3-Phenylacrolein	1.46	0	0.01	97.63	379.02
5	Ethylpyrazine	1.44	0	0.25	46.47	102.57
6	6,7-Dihydro-2,5-dimethylcyclopentapyrazine	1.44	0	—	—	—
7	2,3-Octanedione	1.43	0	0.01	237.48	28.56
8	Phenethyl alcohol	1.43	0	0.56423	129.04	31.33
9	2-Heptanone	1.41	0	0.14	8.92	18.75
10	2,5-Dimethyl-3-n-pentylpyrazine	1.41	0	—	—	—
11	2,3-Dimethylpyrazine	1.39	0	2.5	93.88	309.09
12	Decanoic acid	1.39	0	10	1.44	0.52
13	Dimethyl phthalate	1.39	0	1	7.60	20.04
14	Butyric acid	1.38	0	7.7	23.21	4.46
15	2-Acetyl-5-methylthiophene	1.38	0	—	—	—
16	4-Methylvaleric acid	1.36	0	0.81	26.83	2.63
17	4-Anisaldehyde	1.36	0	—	—	—
18	2-Methylbutyric acid	1.35	0	5.8	7.84	1.08
19	2-Acetylpyrrole	1.35	0	—	—	—
20	p-Cresol	1.35	0	0.039	814.26	123.07
21	Propionic acid	1.34	0	2.19	47.95	5.49

22	Cedrol	1.34	0	0.5	1.77	6.29
23	Hydrocinnamic acid	1.33	0	5	0.46	0.05
24	Phenylacetic acid	1.32	0	12	0.48	0.05
25	Acetylpyrazine	1.31	0	0.01	219.31	127.99
26	1-Butanol	1.30	0	0.1	4.94	15.05
27	2-Propyl-1-pentanol	1.27	0	—	—	—
28	Pentanal	1.25	0	0.012	116.57	295.24
29	Valeric acid	1.25	0	17	5.31	1.31
30	γ-Decalactone	1.22	0	0.1	12.07	4.88
31	Pyrrole	1.19	0	21.9	0.13	0.24
32	2-Methylpyridine	1.17	0.002	—	—	—
33	6-Methyl-5-hepten-2-one	1.17	0.006	—	—	—
34	3,7-Dimethyl-3-octanol	1.16	0.003	—	—	—
35	2-Methyl-3-(2-propenyl)pyrazine	1.15	0	—	—	—
36	2-Vinyl-6-methylpyrazine	1.14	0	—	—	—
37	(E,E)-2,4-Heptadienal	1.12	0.001	0.015	52.04	97.60
38	3,5-Diethyl-2-methylpyrazine	1.11	0	0.16	112.76	38.25
39	2-Acetyl-3-methylthiophene	1.10	0	—	—	—
40	Nonanal	1.08	0	0.035	692.61	885.57
41	Hexanal	1.08	0.001	0.073	158.68	289.83
42	Benzophenone	1.08	0.011	—	—	—
43	β-Carvone	1.06	0.001	0.01	45.41	76.21
44	3-Methylbutanal	1.02	0.001	0.002	303.37	167.72
45	2-Methoxyphenol	1.01	0	—	—	—

Supplementary Figure S1

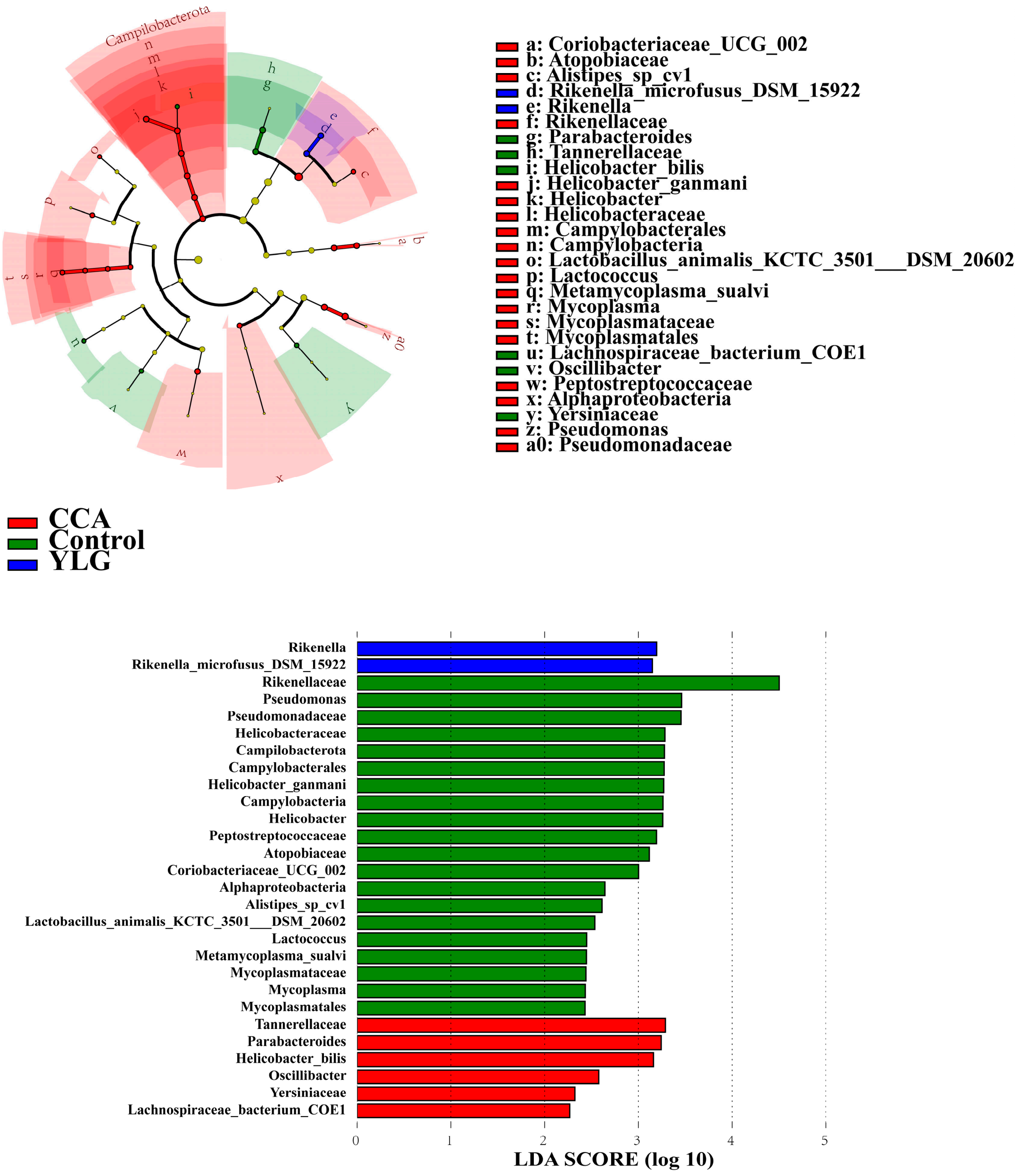

Figure S1. The model microbiomes were identified using LEfSe analysis. Each group had four parallels of mixed samples derived from ten mice represented the results from these parallel samples.
